# Mini-access ascending aorto-bifemoral bypass surgery for the treatment of aortic steno-occlusive disease

**DOI:** 10.1016/j.xjtc.2024.01.006

**Published:** 2024-01-19

**Authors:** Min Jung Ku, Yu Ri Lee, Joon Bum Kim

**Affiliations:** Department of Thoracic and Cardiovascular Surgery, Asan Medical Center, University of Ulsan College of Medicine, Seoul, South Korea

**Keywords:** aortic bypass, minimally invasive surgery, Takayasu arteritis

## Abstract

**Objectives:**

Mid-aortic syndrome is a rare condition characterized by severe aortic narrowing, leading to high upper body blood pressure and organ hypoperfusion, necessitating surgical intervention. Although central bypassing is considered ideal, it involves extensive incisions. To overcome these limitations, less-invasive approaches have been developed. This study aims to introduce a mini-access approach using video-endoscopy and to evaluate the feasibility and outcomes of mini-access ascending aorto-bifemoral bypass surgery.

**Methods:**

From November 2020 to May 2022, we performed ascending aorta to bifemoral artery bypass operations on 7 patients to treat steno-occlusive diseases in the downstream aorta. A Y-graft was created, and procedures were conducted under general anesthesia using video-endoscopy with limited skin incisions.

**Results:**

Intraoperatively, there were no major complications, and none of the patients required cardiopulmonary bypass support. Furthermore, there were no postoperative mortalities or major complications. Postoperatively, the mean ankle-brachial index significantly improved from 0.59/0.59 to 0.96/0.92 (*P* = .004), and the mean glomerular filtration rate increased from 61.1 to 85.3 mL/min/1.73 mm^2^ (*P* = .012). Additionally, symptoms of claudication resolved in all patients.

**Conclusions:**

Videoscope-assisted mini-access aortic bypass surgery not only provides favorable early postoperative outcomes but also represents a technically feasible alternative to traditional surgical approaches for the treatment of steno-occlusive aortic diseases.

## Graphical Abstract

**Figure:**
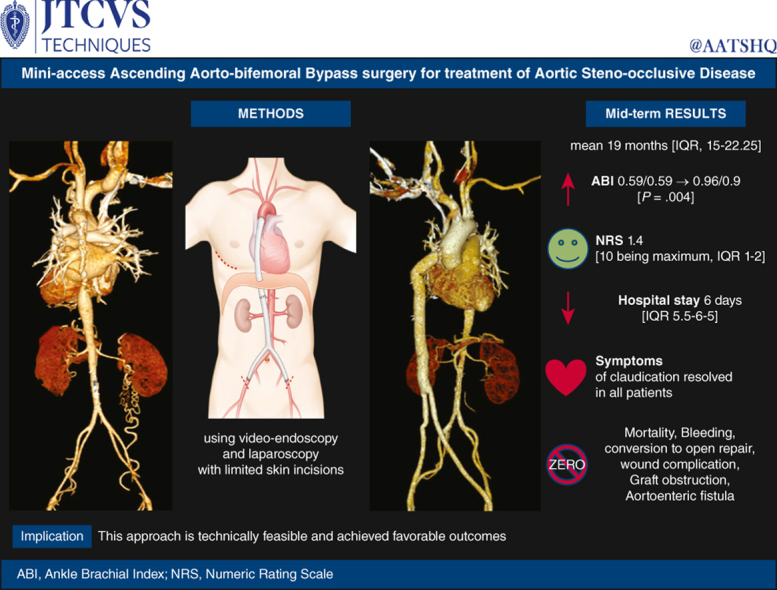
Video-endoscopic mini-access ascending aorta-bifemoral bypass offers patients a less invasive alternative, free from complications.

**Figure:**
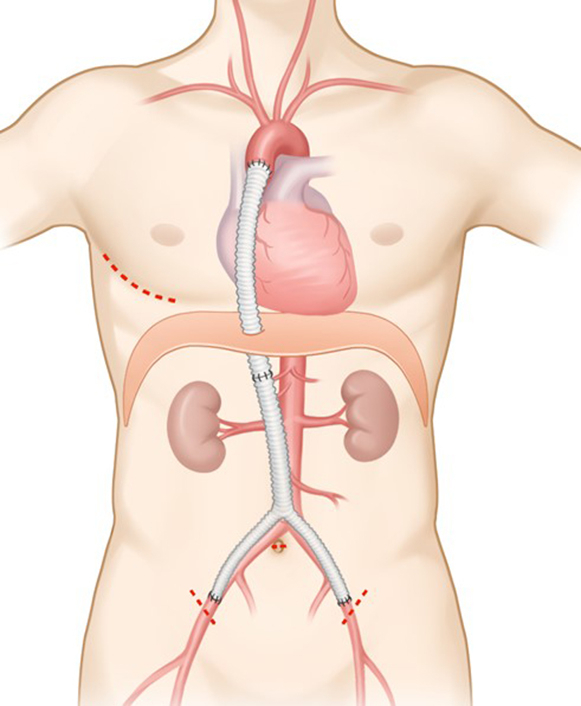
Ascending aorto-bifemoral bypassing using videoscope and laparoscopy.

**Central Message**

Videoscope-assisted mini-access aortic bypass surgery is technically feasible with favorable early outcomes in the treatment of steno-occlusive aortic diseases.

**Perspective**

Effective surgical treatment of steno-aortic occlusive disease necessitates central bypass surgery. Our proposed videoendoscopic mini-access ascending aorta-bifemoral bypass offers patients a less-invasive alternative, free of complications.

Mid-aortic syndrome is a rare clinical condition that is manifested by severe narrowing of the downstream thoracic or abdominal aorta leading to high blood pressure in the upper body and hypoperfusion on visceral organs and lower limbs.^1^^,^^2^ As a result, complications secondary to upper body hypertension, such as stroke and myocardial infarction, often lead to fatal outcomes, whiles long-standing diminished perfusion to the lower body causes chronic renal insufficiency and ischemic symptoms in the mesentery and lower limbs.^2^ Takayasu arteritis is the leading cause of mid-aortic syndrome in adults, but other steno-occlusive disorders of downstream aorta may exhibit similar clinical conditions such as Leriche syndrome, coarctation of aorta, and chronic type B aortic dissection accompanied by severe steno-obstructive true lumen and idiopathic mid-aortic syndrome. Surgical managements are recommended for symptomatic patients with significant pressure differences between upper and lower limbs.^2^, ^3^, ^4^ For this, peripheral bypassing procedures such as axillo-femoral bypassing were a preferred treatment option in the past. With poorer long-term graft patency observed in peripheral bypassing compared with central bypassing, which is explained by the suboptimal inflow source, central bypassing has been suggested as the ideal option to treat this grave disease.^5^, ^6^, ^7^, ^8^ Despite the favorable long-term graft patency, the major limitation of this central bypassing is a need for extensive surgical incisions of combining sternotomy and laparotomy,^5^ which hinder its use in clinical practice.

To overcome this limitation, we have adopted less-invasive approaches to treat mid-aortic syndrome such as limiting the incisions within upper hemi-sternotomy and limited laparotomy.^9^^,^^10^ With accumulating experience, we have come to establish a less-invasive approach, which involves the combined use of thoracoscopy and laparoscopy to establish blood flow between the ascending aorta and bifemoral arteries. We present this mini-access approach for the treatment of mid-aortic syndrome and other aortic occlusive diseases, and share the early outcomes.

## Material and Methods

### Patients

This study was approved by the Institutional Review Board of Asan Medical Center, and informed consent from patients was waived because of the minimal risk of the study inherent to its retrospective observational nature (Institutional Review Board Approval Number: 2022-0910). The study involved consecutive patients undergoing mini-access ascending aorto-bifemoral bypassing by a single surgeon (J.B.K.) in Asan Medical Center, Seoul, Korea, from November 2020 to May 2022.

### Operative Procedures

**Figure 1:**
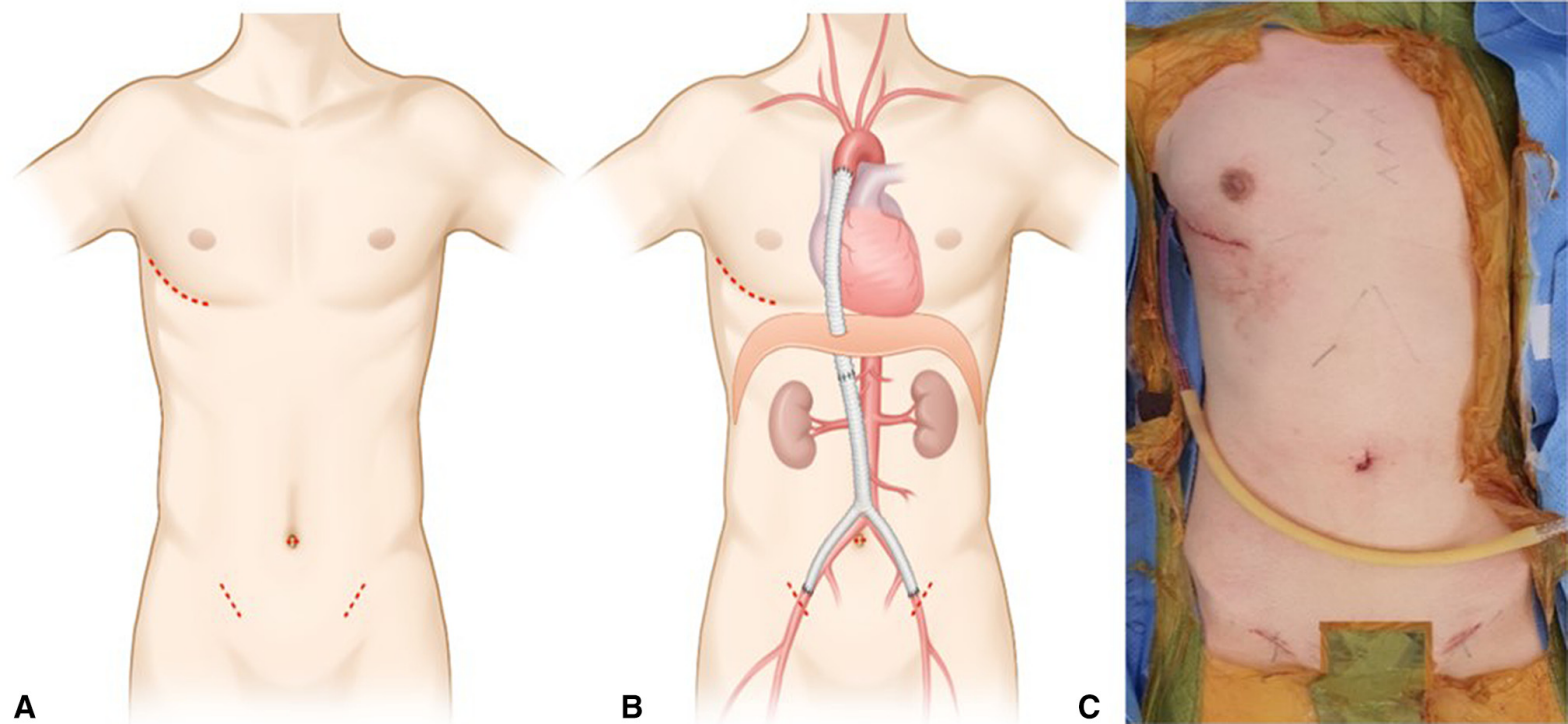
Schematic drawings of the operative plan and an intraoperative picture for Ascending aorto-bifemoral bypassing using videoscope assisted mini-thoracotomy and laparoscopy. A, Limited skin incisions on the right chest, umbilicus and both groins. B, Planning of proximal and distal anastomosis. C, Intraoperative picture after operation with limited skin incisions.

Ascending aorta to bifemoral artery bypass operations were performed on 7 patients with minimal approaches. For surgery, patients were intubated with a double-lumen endotracheal tube under general anesthesia and were prepared by elevating the right side of the trunk (∼15°) in the supine position. During anesthesia, we created a long Y-graft, 18 mm in diameter, by suture connecting between a straight graft and a Y-graft of the same diameter (straight and bifurcated Hemashield grafts; Boston Scientific) to serve as a conduit long enough to cover the distance between the ascending aorta and femoral arteries. After deflating the right lung, a 6-cm mini-thoracotomy was made at the right anterolateral aspect of the chest to enter the fourth intercostal space, and two oblique small (2-3 cm) incisions were made on both groins to expose the common femoral arteries. An additional thoracoscopic port (1 cm) was engaged 3 cm posterior to the main incision, and under thoracoscopic guidance, the ascending aorta was exposed, dissected, and snared after pericardiotomy. Then, we created a laparoscopic port (1 cm) site at the umbilicus. After insufflating CO_2_ gas into the peritoneal space, we created windows at the bilateral femoral sites to enter the peritoneal space under laparoscopic guidance, by which the bifemoral branches of the Y-graft were passed from peritoneal space to femoral sites. Then we created another window at the diaphragm from chest cavity under meticulous cautions not to injure the liver, guided by laparoscopic visualization, which was followed by passing the other end of the graft through this window to enter the thoracic cavity. Then, we examined the entire course of the graft in the peritoneal space and validated free of kinking or torsion of the graft, which should be well seated on the greater omentum. After systemic heparinization, the graft was anastomosed proximally to the mid-ascending aorta first with 4-0 polypropylene running suture under partial aortic clamping. After the release of the aortic clamp, multiple pledgeted reinforcement sutures were made along the anastomotic line. Finally, both femoral arteries were anastomosed using 5-0 polypropylene running sutures under clamping, which was followed by declamping under proper deairing (Figure 1 and Video 1).

### Statistical Analysis

Categorical variables are presented as proportions and percentages. Continuous variables are expressed as the median (interquartile range [IQR]). The comparison of ankle-brachial index (ABI) and glomerular filtration rate (GFR) between preoperative and postoperative results was conducted using the paired Student *t* test or Wilcoxon signed-rank test. All reported *P* values were 2-tailed. IBM SPSS Statistics for Windows, version 21.0 (IBM Corp) was used for statistical analyses.

## Results

**Figure 2:**
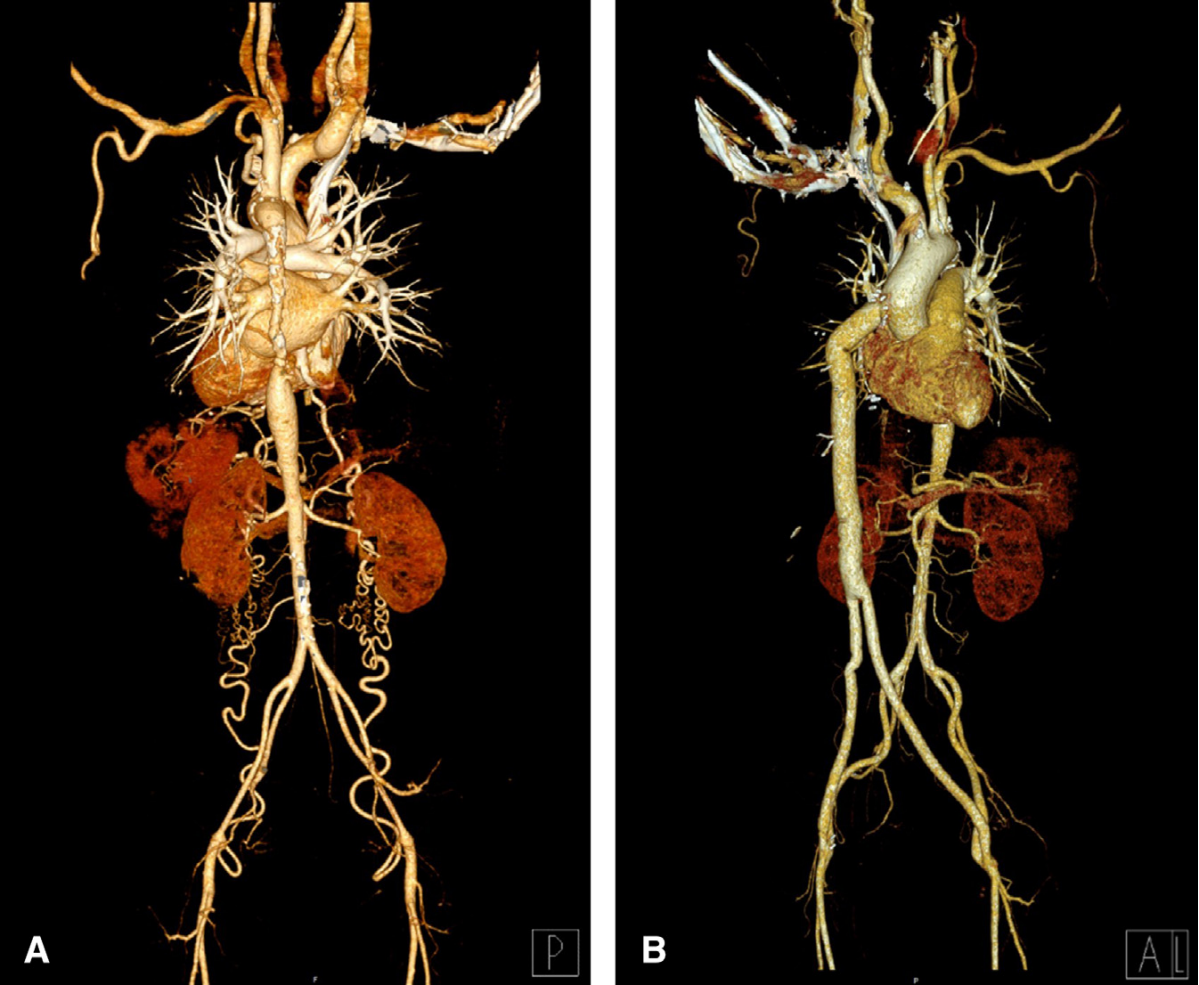
Three-dimensional computed tomography images of patient No. 2. A, Preoperative computed tomography reveals a diffuse narrowing of descending thoracic aorta (minimal diameter: 6.5mm) with multifocal wall calcification observed in the posterior view. B, Postoperative computed tomography reveals a patent bypass graft in the anterior view.

During the study period, 7 patients were enrolled. The baseline characteristics of the patients are detailed in Table 1. Underlying aortic diseases were Takayasu arteritis in 5 patients and aorto-iliac occlusive disease (Leriche syndrome) in 2 patients. All but 1 patient had a severe pressure gradient evidenced by ABI less than 0.6 with accompanying symptoms in the lower limbs. One particular patient, with an ABI of approximately 1.0, had total occlusion of the aorta (Leriche syndrome; case 6), resulting in chronic severe claudication and impotence. Four patients had experienced overt renal insufficiency (GFR < 60 mL/min∗1.72 m^2^, Modification of Diet in Renal Disease equation). Two patients had a history of abdominal surgery. Figure 2 provides a visual representation of three-dimensional computed tomography images, offering a clearer view of the preoperative and postoperative states.

**Table 1:** Patient characteristics and operative profiles

Patient no.	Age (y)	Sex	Diabetes	Preoperative GFR (mL/min/1.73 mm^2^)	Prior abdominal surgery	Etiology	Preoperative ABI (R/L)	Narrowest aortic diameter on CT (mm)
1	51	F		67	Yes	Takayasu	0.57/0.60	6.7
2	57	F		56	Yes	Takayasu	0.33/0.45	7.7
3	50	M		115	-	Leriche	1.1/1.06	Total occlusion
4	48	F		28	-	Takayasu	0.47/0.49	15.3
5	61	F		53	-	Takayasu	0.58/0.55	11.7
6	58	F	Yes	89	-	Leriche	0.62/0.57	Total occlusion
7	59	F		20	-	Takayasu	0.43/0.42	10.3

*GFR*, Glomerular filtration rate; *ABI*, ankle-brachial index; *R*/*L*, right/left; *CT*, computed tomography; *F*, female; *M*, male.

**Figure 3:**
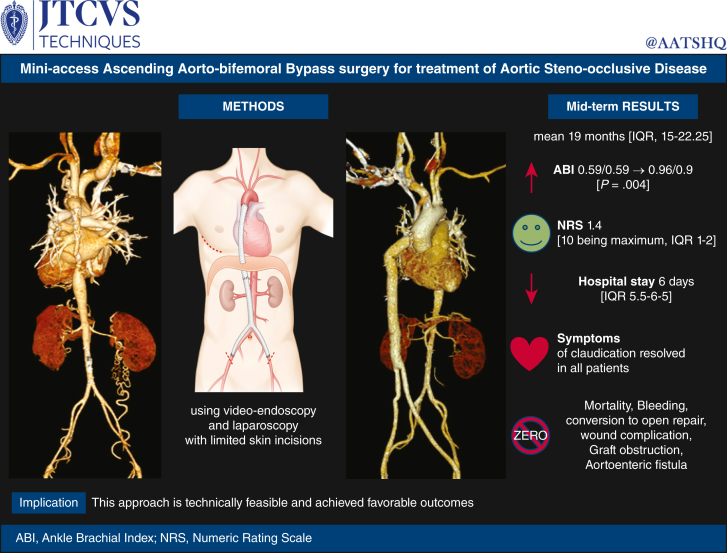
Graphical abstract. Video-endoscopic mini-access ascending aorta-bifemoral bypass offers patients a less invasive alternative, free from complications.

Perioperative parameters are detailed in Tables 2 and 3. There were no cases that required cardiopulmonary bypass support during the procedures. The overall mean procedural time was 216 minutes (IQR, 205-226). Three patients (42.9%) required transfusion of blood products during hospitalization. The median postoperative hospital stay was 6 days (IQR, 5.5-6.5), and the median value of the numeric rating scale on the day of discharge was 1.4 (10 was the maximum; IQR, 1-2). After surgery, the ABI showed normalization or near normalization (0.96/0.92, *P* = .004) in all but 1 patient who showed normal ABI before surgery, and the GFR was improved in 3 of 4 patients with renal insufficiency (*P* = .012). In all patients, symptoms of claudication disappeared postoperatively. Since starting this surgery in 2020, there have been no postoperative deaths, neurologic injuries, reexploration due to bleeding, wound problems, or vascular complications. Additionally, midterm outcomes showed that none of the patients had problems with graft patency or aortoenteric fistula over an average of 19 months (IQR, 15-22.25). Figure 3 shows a Graphical Abstract of the study.

**Table 2:** Procedural time profiles and postoperative course

Variable	Overall (n = 7)
Procedure	
Cardiopulmonary bypass	0 (0%)
Entire procedural time, min	216 (205-226)
Transfusion	
Red blood cell	3 (42%)
Fresh-frozen plasma	2 (28%)
Cryoprecipitate	2 (28%)
Numeric rating scale (maximum = 10)	
At discharge	1.4 (1-2)
Length of stay	
Intensive care unit stay, d	1.4 (1-2)
Postoperative hospitalization stay, d	6 (5.5-6.5)
Follow-up duration (mo)	19 (15-22.25)

Values are number of patients (%) or median [IQR, 25th-75th].

**Table 3:** Comparison values in ankle-brachial index and glomerular filtration rate before and after surgery

Patient no.	Preoperative ABI (R/L)	Postoperative ABI (R/L)	Preoperative GFR	Postoperative GFR
1	0.57/0.60	0.93/0.88	67	118
2	0.33/0.45	0.77/0.81	56	82
3	1.1/1.06	1.0/1.01	115	118
4	0.47/0.49	0.96/0.92	28	45
5	0.58/0.55	1.12/1.12	53	64
6	0.62/0.57	1.1/0.98	89	104
7	0.43/0.42	0.86/0.77	20	66
Median	0.59/0.59	0.96/0.92	61.1 (40.5-78)	85.3 (65-111)
*P* value	.004		.012	

Values are number of patients (%) or median [IQR, 25th-75th]. *ABI*, Ankle-brachial index; *R/L*, right/left; *GFR*, glomerular filtration rate.

## Discussion

Mid-aortic syndrome is a clinical condition caused by the narrowing of the abdominal or descending thoracic aorta, with causes varying from Takayasu arteritis and fibromuscular dysplasia to idiopathic origin,^1^^,^^2^ and surgical treatment is often necessary in severe diseases. Because of the rarity of mid-aortic syndrome and its clinical equivalents, there are limited studies on their natural courses without surgery. Although clear surgical indications are lacking, criteria for coarctation of the aorta are generally applied because of similar hemodynamic consequences. Intervention triggers include aortic narrowing, a pressure gradient of greater than 20 mm Hg between limbs, heart failure, uncontrollable upper-limb hypertension, and lower-body dysfunction.^11^ Although invasive angiography is not routine, ABI is accepted for evaluating pressure gradients.^12^

Although endovascular procedures have improved in the treatment of various aortic disease, their role in this clinical entity has not been established because of the unique nature of constricting aortic narrowing that can hardly be expanded safely by endovascular techniques, leaving the open surgical option as the only effective treatment. Surgical managements for mid-aortic syndrome and other aortic steno-occlusive diseases include a wide variety of options such as patch aortoplasty, replacement of the diseased aorta, and extra-anatomic bypass grafting. Given that the former 2 options necessitate cardiopulmonary bypassing under extensive incisions, the extra-anatomic bypass has been regarded as the most feasible option.^5^^,^^8^ Extra-anatomic bypassing in the treatment of mid-aortic syndrome has been reported as safe and effective for relieving critical ischemic symptoms; however, bypassing between peripheral vessels (ie, axillofemoral) has a limitation of poor long-term graft patency, whereas central bypassing (ie, ascending aorta as the inflow source) usually requires extensive incisions both in the chest and the abdomen.

To address the demand for less-invasive surgery, we have sought to develop a technique using video-endoscopy with limited skin incisions for the treatment of descending thoracic aorta pathologies.^13^ Also, we have previously reported successful cases of aortic bypass through a minimally invasive approach via upper hemi-sternotomy and mini-laparotomy.^9^^,^^10^ Ensuring circumferential control for ascending aorta anastomosis can be challenging because of limited exposure. However, it is crucial for grasping an adequate amount of aortic perimeter. Given the relatively small-sized ascending aorta in these young patients, the safety margin between insufficiently shallow side-biting and excessive clamping leading to shock is narrow; therefore, obtaining this window must be prioritized. Circumferential control in this particular circumstance helps balance the risks of shallow and excessive clamping. Additionally, snaring the aorta provides a safety backup in cases of catastrophic bleeding from the aortotomy site. With careful manipulation under sufficient time, achieving circumferential control has been possible without significant technical challenges, contributing to the safe performance of ascending aorta anastomosis.

Because of the concern of developing fistulation between the gastrointestinal tract and the graft in this technique positioning the graft in the peritoneal space, we seated the peritoneal part of the graft on the omentum so that the graft does not directly contact with visceral organs.^14^

As a result, the outcomes presented in this article are regarded as reasonably acceptable with low rates of transfusions and are expected to show reduced wound problems, less pain, and rapid physical recovery. Contraindications may involve ascending aorta diseases, severe adhesions, conditions intolerant to one-lung ventilation, and unsuitable distal targets. Fortunately, we have not encountered such issues.

### Study Limitations

The experiences presented in this article include all patients referred to surgical therapy given the same diseaseentity during the study period; therefore, the adoption of mini-access surgery was not selective. The study results should be interpreted with caution because they are derived from a high-volume, tertiary referral cardiovascular surgical center that has been highly specialized in minimally invasive surgery for not only valvular diseases but also aortic lesions.^13^^,^^15^^,^^16^ Therefore, the results may not be generalizable to other settings, and there should be steep learning curves to achieve sufficient levels to handle meticulous procedures within limited exposure. In addition, we believe the number of surgical cases shared in the present article is not small given the rarity of illness; however, the experiences should be verified by larger data.

## Conclusions

Mini-access aortic bypass surgery assisted by video-thoracoscopy and laparoscopy may be a reasonable alternative in the management of mid-aortic syndrome or other similar disease entities compared to traditional surgical approaches, demonstrating favorable early postoperative outcomes.

## Conflict of Interest Statement

The authors reported no conflicts of interest.

The *Journal* policy requires editors and reviewers to disclose conflicts of interest and to decline handling or reviewing manuscripts for which they may have a conflict of interest. The editors and reviewers of this article have no conflicts of interest.

## Supplementary Data

Video 1 A 61-year-old female patient (case 5) who was diagnosed with mid-aortic syndrome underwent ascending aorta to bifemoral artery bypass surgery through a minimally invasive approach. The complete details of the procedures and instruments used are shown. Video available at: https://www.jtcvs.org/article/S2666-2507(24)00007-5/fulltext.
