# Supplementary figures and images for: Mini-access ascending aorto-bifemoral bypass surgery for the treatment of aortic steno-occlusive disease
Source: JTCVS Tech. 2024 Jan 19;24:14–9. doi: 10.1016/j.xjtc.2024.01.006 (PMC11145077; doi:10.1016/j.xjtc.2024.01.006)

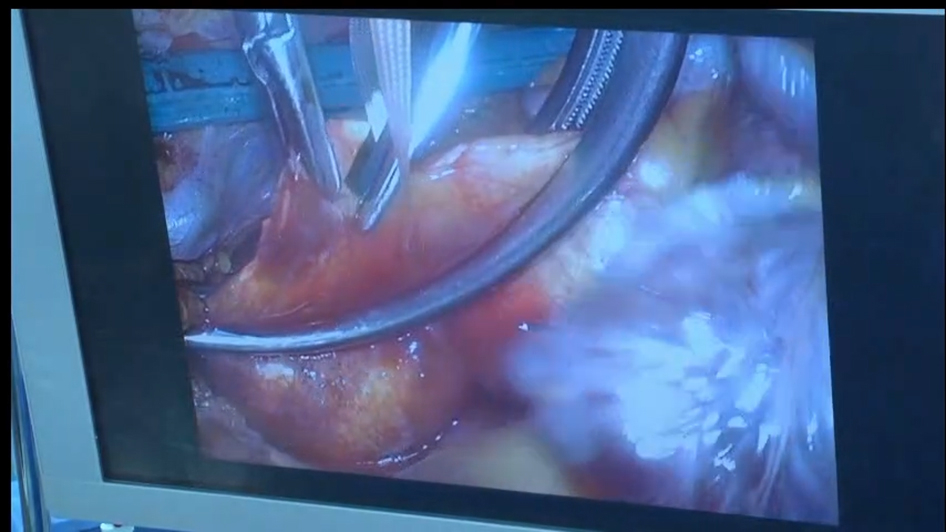

Supplement: Video 1 — A 61-year-old female patient (case 5) who was diagnosed with mid-aortic syndrome underwent ascending aorta to bifemoral artery bypass surgery through a minimally invasive approach. The complete details of the procedures and instruments used are shown. Video available at: https://www.jtcvs.org/article/S2666-2507(24)00007-5/fulltext. [file fx2.jpg]
